# Pilot randomized trial of high- and low-frequency repetitive transcranial magnetic stimulation in post-stroke depression with EEG monitoring

**DOI:** 10.3389/fneur.2025.1671487

**Published:** 2025-10-15

**Authors:** Xiaolin Su, Lin Zhu, Xue Shi, Zian Pei, Brenton Hordacre, Nan Yan, Yi Guo, Ge Dang

**Affiliations:** ^1^Department of Neurology, Shenzhen People’s Hospital (The Second Clinical Medical College, Jinan University, The First Affiliated Hospital, Southern University of Science and Technology, Shenzhen, China; ^2^Department of Electronic and Electrical Engineering, Southern University of Science and Technology, Shenzhen, China; ^3^Innovation Implementation and Clinical Translation (IIMPACT in Health), University of South Australia, Adelaide, SA, Australia; ^4^CAS Key Laboratory of Human-Machine Intelligence-Synergy Systems, Shenzhen Institute of Advanced Technology, Chinese Academy of Sciences, Shenzhen, China; ^5^Guangdong-Hong Kong-Macao Joint Laboratory of Human-Machine Intelligence-Synergy Systems, Shenzhen Institute of Advanced Technology, Chinese Academy of Sciences, Shenzhen, China; ^6^Institute of Neurological and Psychiatric Disorders, Shenzhen Bay Laboratory, Shenzhen, China

**Keywords:** stroke, post-stroke depression, repetitive transcranial magnetic stimulation, resting-state electroencephalography, functional connectivity

## Abstract

**Background:**

Post-stroke depression (PSD) is a prevalent complication that adversely affects recovery following stroke. Repetitive transcranial magnetic stimulation (rTMS) has garnered attention as a potential therapeutic intervention for PSD. This pilot double-blind randomized trial aimed to assess the feasibility and preliminary effects of high- and low-frequency rTMS in PSD, while exploring potential neural mechanisms using electroencephalography.

**Methods:**

Chronic stroke survivors diagnosed with PSD were randomly allocated to receive either high-frequency rTMS targeting the left dorsolateral prefrontal cortex or low-frequency rTMS targeting the right dorsolateral prefrontal cortex for 20 sessions. Hamilton Depression Rating Scale were assessed, and resting-state electroencephalography were recorded at baseline, mid-treatment, and post-treatment.

**Results:**

Both high- and low-frequency rTMS were well tolerated and reduced depressive symptoms at mid- and post-treatment. Electroencephalography analysis did not reveal divergent neural signatures associated with the two protocols. However, altered connectivity linking posterior divisions of the middle frontal gyrus and specific regions in the theta- and beta-band frequencies were associated with the improvement in Hamilton Depression Rating Scale scores.

**Conclusion:**

This pilot study provides preliminary evidence that rTMS is feasible for managing PSD across both high- and low-frequency protocols. EEG analyses suggest potential neurobiological mechanisms, which may inform future research on treatment optimization.

**Clinical trial registration:**

chictr.org.cn, ChiCTR1900021168.

## 1 Introduction

Stroke remains the third-leading cause of death and disability combined globally. The number of stroke survivors is estimated to exceed 200 million by 2050 if current trends persist (1). For effective management of stroke survivors, healthcare providers should not only focus on secondary prevention and rehabilitation, but also on managing mood and emotional disorders that commonly follow stroke. Among the many post-stroke mood and emotional disturbances, post-stroke depression (PSD) is the most prevalent, with a prevalence of about 30%, and is associated with poor recovery, negative quality of life, and increased mortality rates (2). Substantial advancements have been achieved in efficacious interventions for PSD. Beyond pharmacotherapy and psychological therapy for PSD, repetitive transcranial magnetic stimulation (rTMS) has shown therapeutic effects (3).

High-frequency rTMS over the left dorsolateral prefrontal cortex (DLPFC) is a widely used treatment approach in patients with major depressive disorder, whereas low-frequency rTMS over the right DLPFC has demonstrated comparable antidepressant effects (4). Accordingly, a systematic review indicated that both high- and low-frequency rTMS were effective for patients with PSD (5). However, the majority of previous low-frequency rTMS studies were performed alongside antidepressant treatment (6). A randomized trial directly comparing the effects of high- and low-frequency rTMS on PSD has not yet been reported. Low-frequency rTMS has garnered clinical interest for the treatment of PSD for various reasons. Epilepsy occurs in 6.4–15% stroke survivors (7). Nevertheless, rTMS-induced seizure is a rare but serious adverse event, most frequently occurs during high-frequency rTMS (8). Low-frequency rTMS, thought to down regulate cortical activity, is known for its safety in patients with epilepsy (9), and might be safer for individuals with PSD. If therapeutic efficacy is comparable, low-frequency rTMS over the right DLPFC might be the preferred treatment due to its greater safety profile.

Although the neural mechanisms underlying the therapeutic efficacy of rTMS remain unclear, connectivity changes are considered crucial for mediating rTMS-induced depression relief in depressive disorder (10). Numerous functional magnetic resonance imaging studies reported alterations of resting-state functional connectivity following rTMS treatment over the DLPFC (11). Electroencephalography (EEG), characterized by high temporal resolution and frequency specific information, offers unique insights in brain activity (12). Advances in EEG source localization, partially mitigating low spatial resolution, enhance its utility for studying brain networks (13). Regarding resting-state EEG connectivity changes following rTMS treatment, alterations in the theta, beta, and gamma band frequencies have been reported in previous major depressive disorder studies (14–16). In a recent PSD study, increased theta-band EEG functional connectivity between the left frontal and right parietal cortices was observed following high-frequency rTMS applied over the left DLPFC, coinciding with the amelioration of depression symptoms (17). The investigation of functional connectivity changes may be beneficial for optimizing the rTMS strategy for PSD.

This pilot study aimed to assess the feasibility and preliminary effects of high-frequency rTMS over the left DLPFC and low-frequency rTMS over the right DLPFC in patients with PSD without concomitant antidepressant treatment. Additionally, we hypothesized that the reduction in depressive symptoms following rTMS treatment would be associated with changes in EEG functional connectivity.

## 2 Materials and methods

### 2.1 Participants and study design

This study followed CONSORT recommendations (18) and was registered in the Chinese Clinical Trial Registry (ChiCTR1900021168). The study was approved by the ethics committee of Shenzhen People’s Hospital, and consent forms were signed by all participants. The inclusion criteria were as follows: age 18–75 years; chronic stroke survivors of more than 3 months from the first stroke episode and without a second stroke; the depressive symptoms occurred more than one month following the stroke; met the *Diagnostic and Statistical Manual of Mental Disorders, Fifth Edition* diagnosis of “mood disorder due to another medical condition (stroke) with major depressive-like episode”; 24-item Hamilton Depression Rating Scale (HAMD-24) score ≥20; Chinese speaking; and self-reported right-handedness. Patients taking any anti-depression drug 4 weeks prior to enrollment, with Mini-Mental Status Exam (MMSE) score <18, with other psychiatric history (e.g., schizophrenia), with other neurological diseases (e.g., Alzheimer’s disease), with severe medical conditions (e.g., heart failure), with a history of substance or alcohol abuse, or with any contraindication to TMS (19) were excluded.

This study was designed as a double-blind, randomized controlled trial. Participants were randomly allocated to either the high-frequency rTMS group targeting the left DLPFC (HF-left) or the low-frequency rTMS group targeting the right DLPFC (LF-right). The allocation sequence was generated using a random number generator by an independent assessor uninvolved in stimulation or analysis. Participants, outcome assessors and data analyzers were kept unaware of the group allocation. Baseline evaluations encompassed demographic data, clinical features, HAMD scores, MMSE scores, National Institutes of Health Stroke Scale (NIHSS) scores, and resting-state EEG (rsEEG) measurements (labeled as timepoint 0, T0). Subsequently, the participants completed 20 daily sessions of rTMS (5 days a week, over a 4-week period). HAMD scores and rsEEG assessments were repeated upon completion of 10 and 20 rTMS sessions, denoted as timepoints 1 (T1, mid-treatment, after 10 sessions) and 2 (T2, post-treatment, after 20 sessions), respectively.

### 2.2 Repetitive transcranial magnetic stimulation

rTMS was delivered via a MagPro ×100 magnetic stimulator connecting a B658 coil (MagVenture, Farum, Denmark). The coil was situated tangentially on the scalp, with its handle positioned diagonally toward the posterior-lateral direction at a 45-degree angle from the midline. The resting motor threshold was determined as the lowest stimulus intensity required to elicit a motor-evoked potential surpassing 50 mV in at least 5 out of 10 trials within the relaxed abductor pollicis brevis muscle (20). For HF-left rTMS, the coil was positioned over the F3 electrode site of the 10–10 EEG system to target the left DLPFC. Pulses were delivered at 10 Hz with 100% intensity of the resting motor threshold (4-s trains, 26-s intertrain interval, 3,000 pulses/session). For LF-right rTMS, the coil was situated over the F4 electrode site. Pulses were administered at 1 Hz with 100% intensity of the resting motor threshold (10-s trains, 1-s intertrain interval, 2,100 pulses/session).

### 2.3 EEG acquisition and preprocessing

For the EEG recordings, patients sat comfortably relaxed in a chair. Sixty-four Ag–AgCl monopolar electrodes were positioned according to the standard 10–10 system., and impedances <10 kΩ were maintained. EEG signals were recorded for 8 min with eyes closed. A BrainAmpDC amplifier (Brain Products GmbH, Gilching, Germany) was used. The FCz channel served as the reference electrode, with the ground electrode positioned at AFz during EEG recording. The signals were initially recorded at a sampling rate of 5,000 Hz, and subsequent offline analysis was conducted. EEG data were exported to MATLAB (MathWorks, Inc., Natick, MA, USA), where preprocessing was carried out using the EEGLAB toolbox (21). First, visible contaminated segments were manually removed. The data underwent filtering between 1 and 45 Hz using a finite impulse response filter. Bad channels were eliminated and interpolated through spherical spline interpolation. Subsequently, the data were segmented into 2-s epochs. Artifacts such as eye blinks, muscle activity, and heart noise were eliminated by an independent component analysis approach. Finally, the signals were referenced to the common average and filtered into delta (1–3 Hz), theta (4–7 Hz), alpha (8–12 Hz), and beta (13–30 Hz) frequency bands.

### 2.4 EEG source connectivity analysis

We used the Brainstorm toolbox for EEG source localization (22). A FreeSurfer average brain template was employed for 62 EEG channels (without FCz and AFz) co-registration. The head model was computed using a symmetric boundary element method through OpenMEEG (23). No noise modeling was used as noise covariance. The weighted minimum norm estimate algorithm was employed as an inverse solution. Unconstrained dipoles at 3003 vertices on the cortical surface were generated and the current density was measured. In the Montreal Neurological Institute space, the cortical surface was parcellated into 31 regions of interest (ROIs), derived from the parcellation of resting-state functional magnetic resonance imaging in a prior study (24). The debiased weighted phase lag index (dwPLI) was computed to quantify EEG functional connectivity (25). It serves as an index of phase-synchronization, with a range between 0 and 1, wherein higher values signify stronger connectivity.

### 2.5 Partial least squares regression

The study utilized the N-way Toolbox (26) to perform the partial least squares (PLS) regression analyses which is appropriate to investigate the relationship between brain activity and behavior in neuroimaging research (27). The left and right posterior divisions of the middle frontal gyrus (LPMFG and RPMFG), approximating to the stimulated left or right DLPFC, were employed as separate seed regions of interest. The changes in dwPLI values between the PMFG to other ROIs, from T0 to T1 and from T0 to T2 within each frequency band, were considered separate independent variables. Correspondingly, the relative changes in HAMD scores from T0 to T1 and from T0 to T2 were designated as the dependent variables. PLS identified a model with a threshold set at 0.75. Cross-validation was conducted employing the leave-one-out prediction method. The mean connectivity changes within the network linking the PMFG and the ROIs identified in the PLS models were each correlated with the relative change in HAMD scores. Bonferroni correction was applied for multiple comparisons.

### 2.6 Statistical analyses

Statistical analyses were conducted using R Statistical Software (v4.1.2; R Core Team 2021). The Shapiro–Wilk test was used to assess normality. Nonparametric statistics were used as required. For the comparison of demographics and clinical features between HF-left and LF-right groups, independent *t*-tests, Fisher’s Exact tests or Chi-square test were employed. To assess changes in HAMD scores over time, a two-way repeated measures ANOVA was conducted, with time (T0, T1, and T2) as within-subject factors and group (HF-left and LF-right) as a between-subject factor, followed by post-hoc testing using Bonferroni corrected *t*-tests. EEG functional connectivity matrices were compared between groups using network-based statistics (NBS) implemented via the NBS toolbox (28). All analyses were performed in the modified intention-to-treat population, including participants who received at least one treatment.

## 3 Results

### 3.1 Participant characteristics

**Figure 1:**
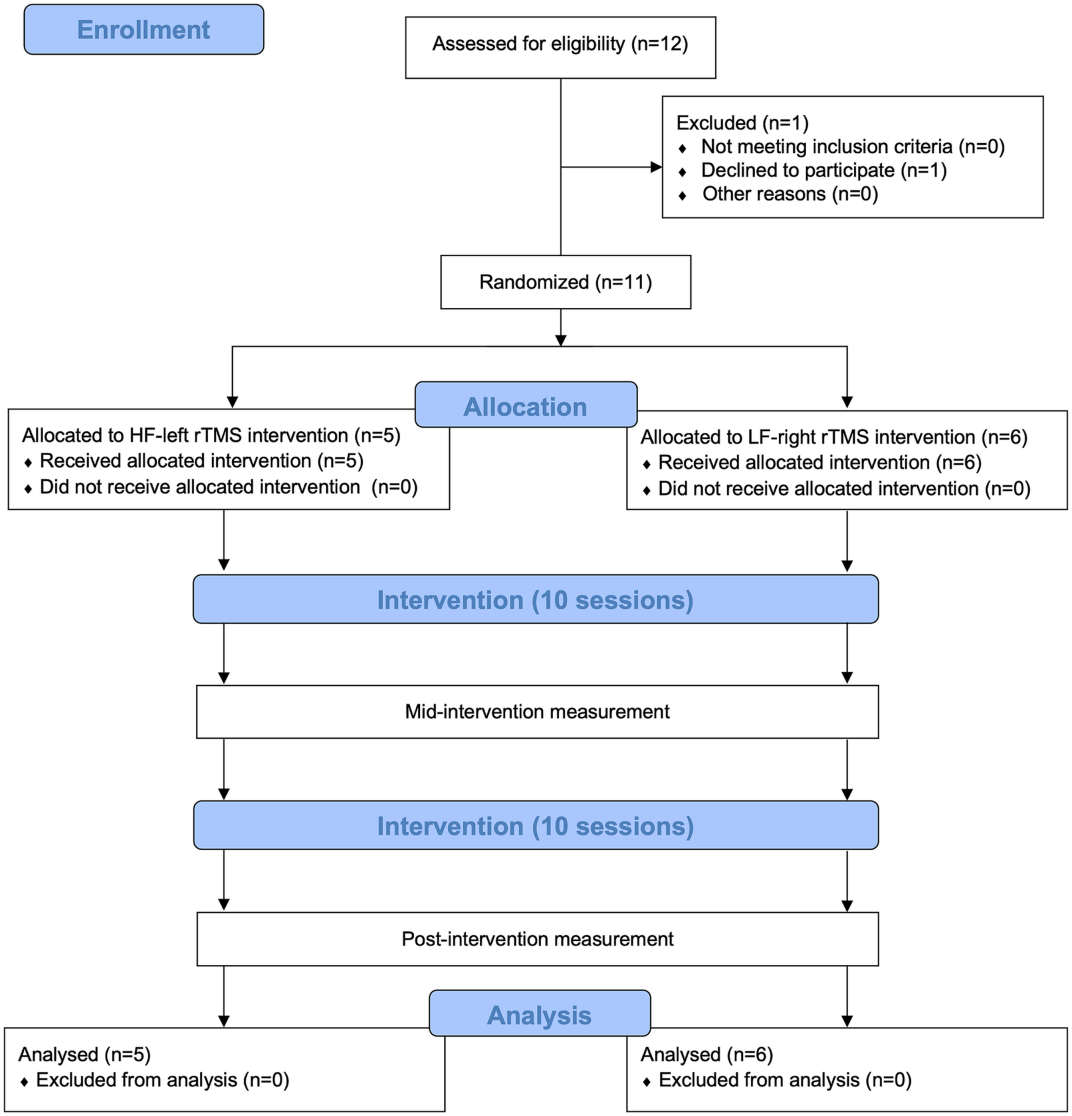
Flowchart depicting the CONSORT guidelines for the study.

Figure 1 presents the participant enrollment in the study. Twelve patients were enrolled, and one withdrew for personal reasons before randomization. Five patients were randomized to the HF-left rTMS group and six were randomized to the LF-right rTMS group. They completed the rsEEG recordings and neuropsychological assessments at T0, T1, and T2. Table 1 displays the demographic and baseline characteristics. No significant differences in age, sex, baseline HAMD, MMSE, NIHSS scores, lesion hemisphere and location, and time since stroke were observed across the groups (all *p* > 0.05). There was no significant correlation between HAMD and MMSE, or NIHSS score (all *p* > 0.05).

**Table 1:** Demographic and clinical characteristics of the participants in high-frequency rTMS targeting the left dorsolateral prefrontal cortex (HF-left) and low-frequency rTMS targeting the right dorsolateral prefrontal cortex (LF-right) groups.

Characteristic	HF-left (*n* = 5)	LF-right (*n* = 6)	*p*-value
Age (years)	58.6 ± 5.3	61.8 ± 9.3	0.51
Sex, female [*n* (%)]	2 (40%)	4 (67%)	0.57
HAMD	24.2 ± 5.4	26 ± 9.19	0.71
MMSE	26.2 ± 4.09	26.2 ± 4.71	0.99
NIHSS	1 ± 1.73	2.5 ± 2.74	0.32
Lesioned hemisphere (n)			0.18
Right	5	3	
Left	0	2	
Both	0	1	
Lesion Location (*n*)			0.56
Sub-cortical	4	3	
Cortical-sub-cortical	0	1	
Brainstem	1	1	
Corpus callosum	0	1	
Time since stroke (months)	9.4 ± 4.4	13.8 ± 8.7	0.33

HAMD, Hamilton Depression Rating Scale. MMSE, Mini-Mental Status Exam. NIHSS, National Institutes of Health Stroke Scale.

### 3.2 Treatment responses

**Figure 2:**
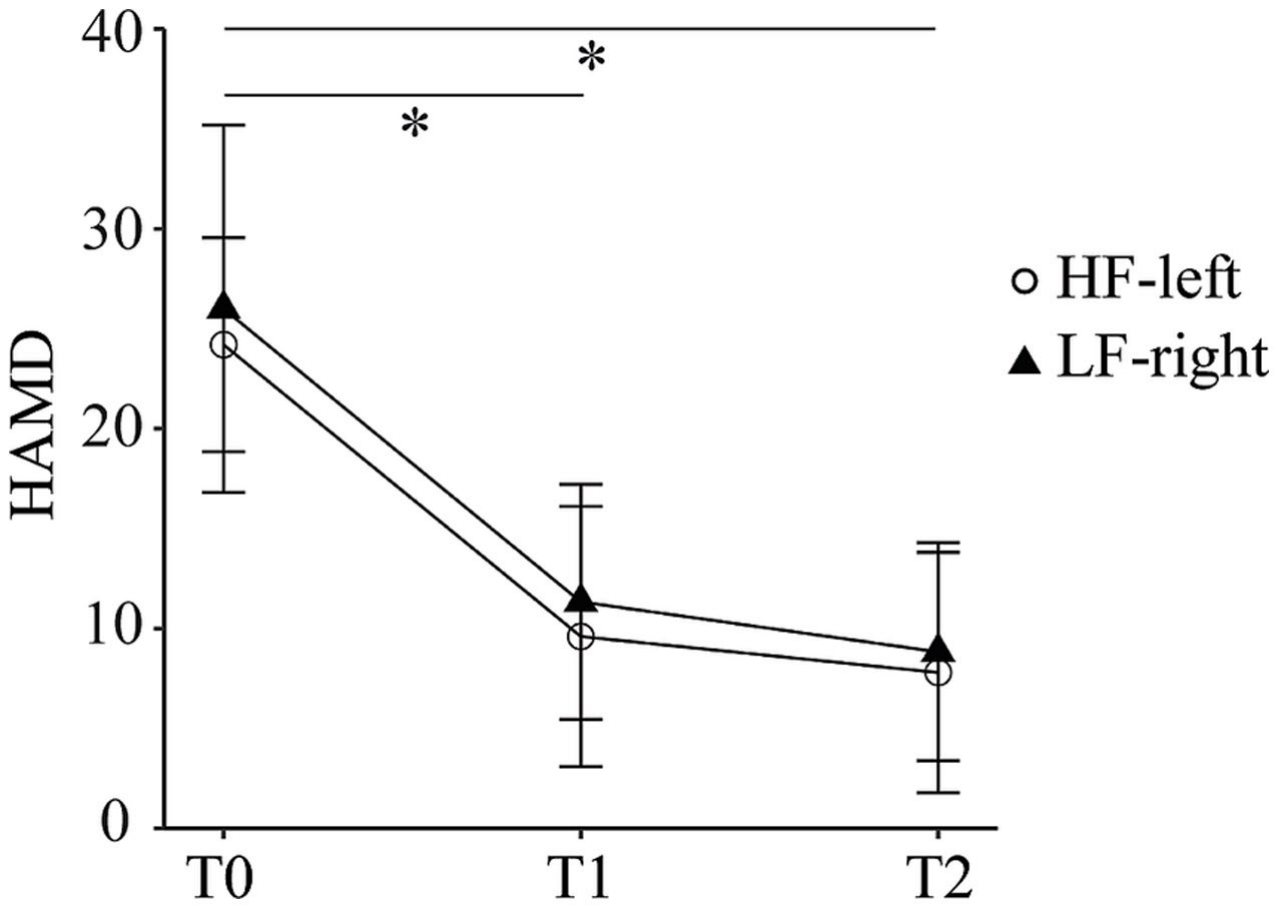
Hamilton Depression Rating Scale scores at baseline (T0), mid-treatment (T1), and post-treatment (T2) in the high-frequency left dorsolateral prefrontal cortex (HF-left) and low-frequency right dorsolateral prefrontal cortex (LF-right) rTMS groups.

Both HF-left and LF-right rTMS groups showed reductions in HAMD scores over time. Mean scores decreased from 24.2 ± 5.4 at baseline to 9.6 ± 6.5 at T1 and 7.8 ± 6.02 at T2 in the HF-left group (Cohen’s *d* = 2.42, 95% CI: 0.52–4.32 and 2.87, 95% CI: 0.70–5.03, respectively), and from 26 ± 9.19 to 11.3 ± 5.89 and 8.83 ± 5.46 in the LF-right group (*d* = 1.73, 95% CI: 0.60–2.87 and 2.26, 95% CI: −0.09 - 4.61) (Figure 2). The ANOVA revealed no significant main effect of group or group × time interaction, but a significant main effect of time was observed (*F*_2,8_ = 69.08, *p* < 0.001). The *post hoc* analysis uncovered a notable reduction in HAMD scores from T0 to T1 (*t*_10_ = −8.22, *p* < 0.001, Bonferroni-corrected), as well as from T0 to T2 (*t*_10_ = −6.90, *p* < 0.001, Bonferroni-corrected). Both interventions were well-tolerated and safe, with full adherence and no reported adverse events.

### 3.3 EEG differences between groups

NBS were used to assess functional connectivity across all frequency bands at T0, T1, and T2. No significant between-group differences or within-group changes were observed (primary *t*-threshold = 0.05, 5,000 permutations, family-wise error corrected *p* > 0.05).

### 3.4 EEG changes and clinical improvements

**Figure 3:**
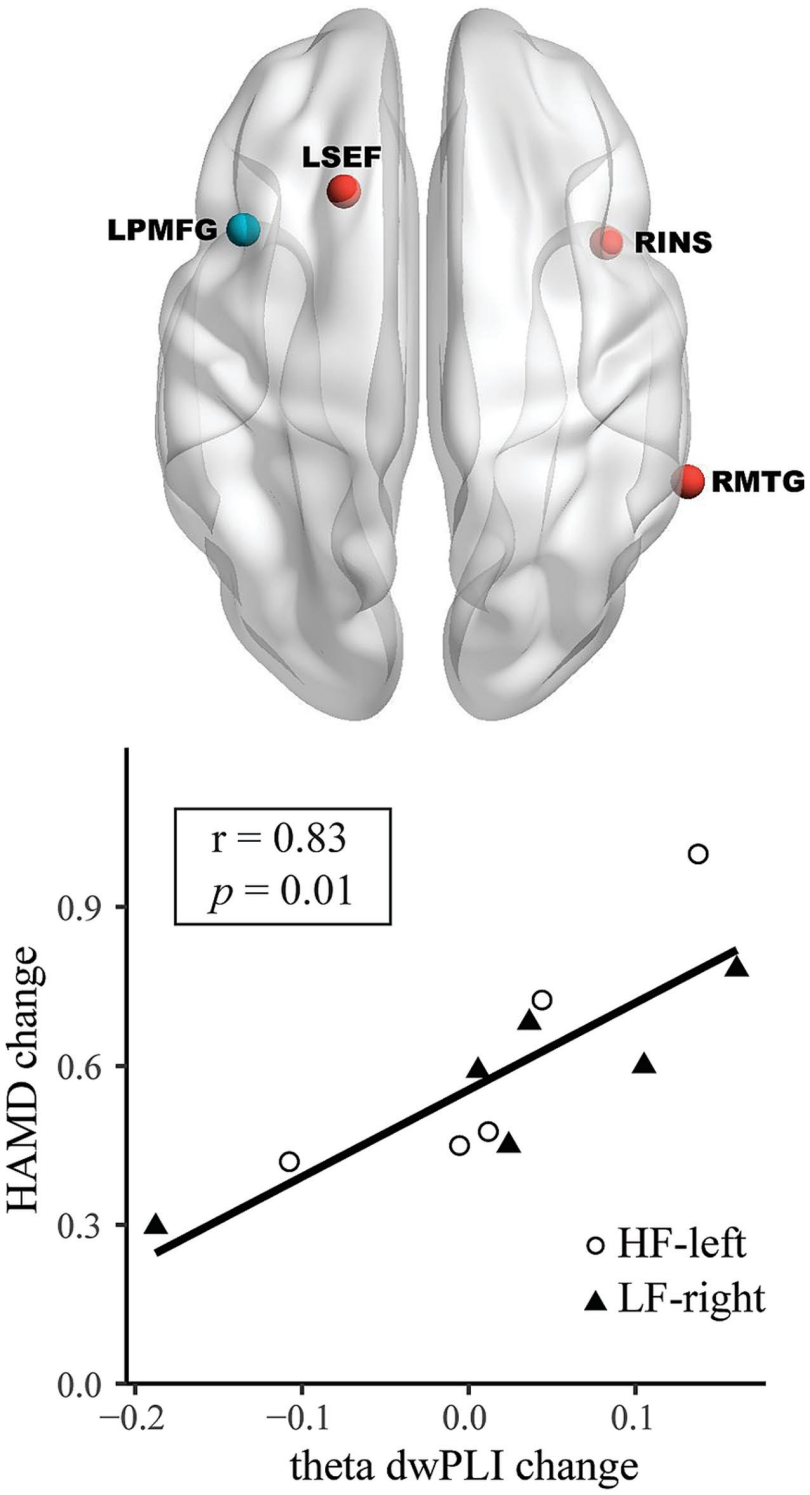
From baseline (T0) to mid-treatment (T1), changes in left posterior division of middle frontal gyrus (LPMFG) connectivity, correspond with clinical response in the theta band. Connectivity alterations in theta band, involving LPMFG and distinct brain regions, correlate with improvements in Hamilton Depression Rating Scale (HAMD) scores. Lower panels depict the correlation between mean connectivity changes in the network and HAMD score changes in high-frequency rTMS targeting the left dorsolateral prefrontal cortex (HF-left) and low-frequency rTMS targeting the right dorsolateral prefrontal cortex (LF-right) groups. LSEF, left supplementary eye field; RINS, right insular cortex; RMTG, right middle temporal gyrus.

For HAMD changes from T0 to T1, a theta frequency PLS model identified a connection between the LPMFG and the left supplementary eye field (LSEF), right insular cortex (RINS), and right middle temporal gyrus (RMTG); a higher theta band dwPLI change in this model correlated with a greater HAMD score change (*r* = 0.83, 95% CI: 0.46–0.96, Bonferroni-corrected *p* = 0.01) (Table 2 and Figure 3). The PLS models were not significant in the delta, alpha, and beta bands for the LPMFG, nor in any frequency band for the RPMFG (all *p* > 0.05; Table 2).

**Table 2:** Fitted R^2^ and cross-validated R^2^ of PLS models generated for rTMS response and connectivity change in different frequency bands from baseline to mid-treatment (T0-T1), and from baseline to post-treatment (T0-T2).

Frequency	T0-T1		T0-T2	
	LPMFG	RPMFG	LPMFG	RPMFG
Delta	[0.35; 0.31]	[0.45; 0.44]	[0.35; 0.34]	[0.38; 0.26]
Theta	[0.59; 0.53]*	[0.35; 0.31]	[0.32; 0.30] *	[0.74; 0.50]
Alpha	[0.76; 0.62]	[0.49; 0.45]	[0.51; 0.49]	[0.58; 0.54]
Beta	[0.62; 0.52]	[0.60; 0.37]	[0.30; 0.13]	[0.36; 0.35] *

Data are presented as [Fitted R^2^; Cross-validated R^2^]. *Statistically significant results (Bonferroni-corrected *p* < 0.05). LPMFG, left posterior division of middle frontal gyrus. RPMFG, right posterior division of middle frontal gyrus.

**Figure 4:**
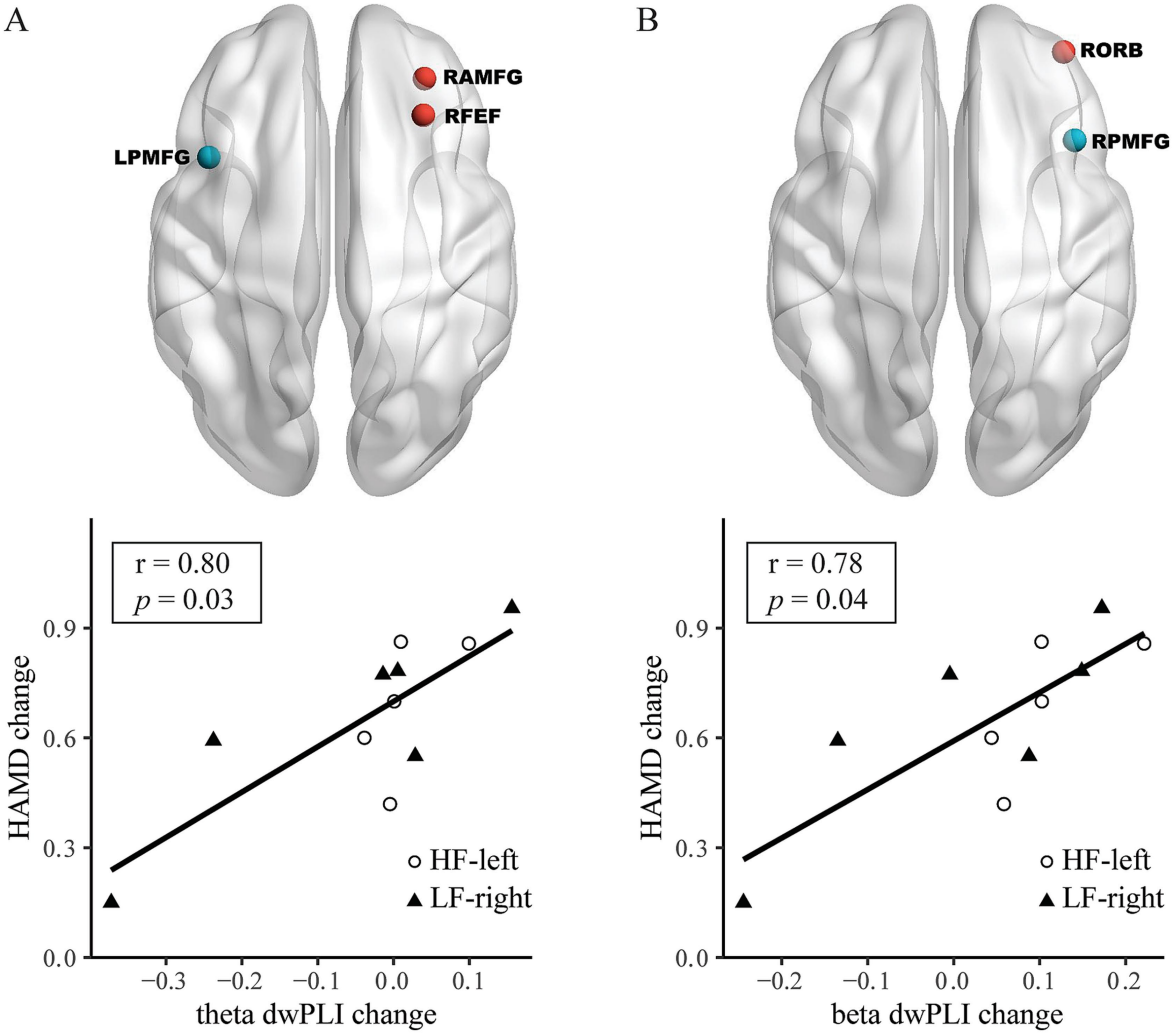
From baseline (T0) to post-treatment (T2), alterations in both left posterior division of middle frontal gyrus (LPMFG) and right posterior division of middle frontal gyrus (RPMFG) connectivity, are associated with clinical response across different frequency bands. Connectivity alterations in LPMFG connectivity in theta **(A)** band, as well as RPMFG connectivity in beta **(B)** band involving distinct brain regions, correlate with improvements in Hamilton Depression Rating Scale (HAMD) scores. Lower panels depict the correlation between mean connectivity changes in each network and HAMD score changes in high-frequency rTMS targeting the left dorsolateral prefrontal cortex (HF-left) and low-frequency rTMS targeting the right dorsolateral prefrontal cortex (LF-right) groups. RAMFG, right anterior division of middle frontal gyrus; RFEF, right frontal eye field; RORB, right orbitofrontal cortex.

For HAMD changes from T0 to T2, a theta frequency PLS model identified the connection between the LPMFG and right anterior division of middle frontal gyrus (RAMFG) and right frontal eye field (RFEF); a larger theta band dwPLI change was correlated with a greater HAMD score change (*r* = 0.80, 95% CI: 0.37–0.94, Bonferroni-corrected *p* = 0.03) (Table 2 and Figure 4A). A beta frequency PLS model identified the connection between the RPMFG and the right orbitofrontal cortex (RORB); a greater beta band dwPLI change was correlated with a higher HAMD score change (*r* = 0.78, 95% CI: 0.34–0.94, *p* = 0.04, Bonferroni-corrected) (Table 2 and Figure 4B). PLS models for LPMFG in the delta, alpha, and beta were not significant, as well as that for RPMFG in the delta, theta and alpha band (all *p* > 0.05, Table 2).

## 4 Discussion

This pilot study suggests that high-frequency rTMS to the left DLPFC and low-frequency rTMS to the right DLPFC are safe, feasible, and potentially effective for reducing depressive symptoms in PSD, with generally similar clinical outcomes between the two treatments. Moreover, rTMS-induced clinical improvements may correlate with changes in theta- and beta-band EEG functional connectivity following treatment. After ten sessions, increased theta-band connectivity of the LPMFG with the LSEF, RINS, and RMTG correlated with reductions in HAMD scores. After 20 sessions, HAMD score changes were linked to theta-band connectivity of LPMFG with RAMFG and RFEF, and to beta-band connectivity between RPMFG and RORB.

Preliminary results suggest that low-frequency right DLPFC rTMS and high-frequency left DLPFC rTMS produce comparable antidepressant effects in PSD, consistent with findings in MDD treatment (29). High-frequency rTMS over the left DLPFC has previously been reported to alleviate depressive symptoms in PSD compared with sham stimulation (17). As stroke is the most common cause of epilepsy in adults (7), low-frequency rTMS, which is safe for epilepsy, is preferred for stroke survivors. Previous studies have reported that low-frequency rTMS combined with antidepressants improves depressive symptoms in PSD. However, the distinct side-effect profile of antidepressants may compromise tolerability, complicate therapy, or necessitate discontinuation (30). For example, fluoxetine appears effective in alleviating depression in PSD but may pose risks, including fractures, hyponatremia, and possible impairments in memory and communication (31). Our findings highlight the potential utility of low-frequency rTMS alone. Along with feasible recruitment, reliable outcomes, and no adverse events support its safety, tolerability, and the rationale for a larger confirmatory trial.

Differing neurophysiological responses were considered to underpin high-frequency and low-frequency rTMS. Generally, high-frequency rTMS leads to increased excitability, whereas low-frequency rTMS results in cortical inhibition when applied to the motor and prefrontal cortices (32). As rTMS over the DLPFC has been extensively reported to modulate resting-state functional connectivity (11), we further explored connectivity alterations associated with treatment response. Due to the lack of significant group differences in functional connectivity and the small sample size, we focused on a shared mechanism by which both high- and low-frequency rTMS alleviate depressive symptoms, rather than on their differences. A preliminary finding of this study was that rTMS ameliorates depressive symptoms, accompanied by enhancing functional connectivity in the theta and beta frequency bands.

Theta oscillation, a main feature dominating the resting-state EEG, arises from complex interactions between the medial septum-diagonal band of Broca and the intra-hippocampal circuits (33). Theta oscillation involves various aspects of cognition such as memory encoding, locomotion, and spatial navigation (34). Theta power has also been proposed as a potential biomarker for depression, with observed alterations in the anterior cingulate cortex, fronto-midline, and frontal theta power (35). Impaired theta connectivity in anterior regions in patients with major depression were reported (36). Theta connectivity alterations were observed in treatment responders of rTMS therapy in depression patients; nevertheless, the contradictory outcomes from a larger dataset were reported by the same group of authors recently (14, 37). In stroke survivors, functional connectivity in the theta band were significantly increased after intermittent theta burst stimulation (38). Moreover, the interhemispheric theta frontoparietal connectivity may be a mechanism underlying the effectiveness of high-frequency rTMS in PSD (17).

Beta oscillations, primarily generated in the cortex and basal ganglia, are thought to support sensorimotor, cognitive, and affective processes (39). Alterations in beta-band connectivity have been widely reported in depression, with both increases and decreases observed (40). Although attenuation of beta-band EEG connectivity is frequently reported in stroke (41, 42), its relationship with post-stroke depression remains unclear. High-frequency left prefrontal rTMS has been shown to increase resting-state beta-band connectivity between the left dorsolateral prefrontal cortex and limbic regions in treatment-resistant depression (15). Consistent with this, we found that increases in beta-band connectivity were positively correlated with the change in HAMD scores following rTMS treatment. How functional connectivity relates to rTMS-induced clinical improvement in PSD warrants further investigation.

This study is subject to several limitations warranting consideration. The most important is the very small sample size, which restricts the ability to draw firm conclusions about treatment effects. Additionally, the lack of a sham control group limits the ability to distinguish specific from placebo effects, particularly since no significant differences between groups were observed here. The absence of long-term follow-up prevents assessment of the durability of improvements. The study did not track stroke severity during treatment, restricting understanding of the intervention’s impact on overall disease progression. Moreover, while functional connectivity was examined as an exploratory feature, the small sample precludes meaningful interpretation of these findings. Finally, the lack of analysis of lesion location and volume limits interpretation of connectivity changes and restricts insights into how specific brain regions may influence treatment response. These limitations necessitate a larger, adequately powered trial to assess efficacy and the mechanistic role of functional connectivity.

## 5 Conclusion

In conclusion, this pilot study demonstrates that low-frequency rTMS is feasible, well tolerated, and may have effects comparable to high-frequency rTMS in alleviating depressive symptoms in patients with PSD, with recruitment, adherence, and data collection successfully achieved. These findings provide valuable guidance for designing a larger, adequately powered trial. Additionally, exploratory analyses suggest that theta- and beta- EEG functional connectivity may provide insights into treatment mechanisms, warranting further investigation in larger trials.

## Data Availability

The raw data supporting the conclusions of this article will be made available by the authors, without undue reservation.
